# Post-mortem Diagnosis of Heme Oxygenase-1 Deficiency by Whole Exome Sequencing in an Iranian Child

**DOI:** 10.22088/IJMCM.BUMS.8.4.300

**Published:** 2019

**Authors:** Fatemeh Tahghighi, Nima Parvaneh, Vahid Ziaee

**Affiliations:** 1 *Children's Medical Center, Pediatrics Center of Excellence, Tehran, Iran.*; 2 *Department of Pediatrics, Tehran University of Medical Sciences,Tehran, Iran.*; 3 *Division of Allergy and Clinical immunology, Department of Pediatrics, Tehran University of Medical Sciences,Tehran, Iran.*; 4 *Research Center for Immunodeficiencies, Tehran University of Medical Sciences, Tehran, Iran.*; 5 *Pediatric Rheumatology Research Group, Rheumatology Research Center, Tehran University of Medical Sciences, Tehran, Iran.*

**Keywords:** Heme oxygenase-1 deficiency, post-mortem diagnosis, HO-1 gene, Iranian child

## Abstract

Heme oxygenase-1 (HO-1) is an inducible enzyme involved in the catalysis of heme conversion into biliverdin. We describe a patient with a novel stop-gain mutation in the *HMOX1* coding sequence resulting in HO-1 deficiency. A 17-month-old female with fever, tachypnea, and signs of respiratory distress was referred to our center. Four admissions ensued during the eight months follow up. At the first admission, she had massive pericardial effusion without any laboratory findings for tuberculosis, viral infectionsor malignancies.An abdominal ultrasound examination confirmed hepatomegaly.Laboratory findings showed leukocytosis, thrombocytosis, hemolytic anemia, elevated inflammatory markers, increased levels of the hepatic transferase, triglycerides and ferritin as well as decreased level of fibrinogen. Other laboratory investigations were negative blood cultures, normal bone marrow aspiration, and normal serology viral infections. Immunodeficiency and auto-inflammatory syndromes were ruled out. Hepatic biopsy showed iron deposits. The patient was initiated on corticosteroids; however, her clinical condition was progressively deteriorated, and she died of recurrent fever, bleeding, heart failure, and ascites. Post-mortem whole exome sequencing revealed a homozygous mutation (exon3: c.A610T, p.K204X) on the *HMOX1* gene. The parents were found to be heterozygous for that mutation. The laboratory findings and clinical features of our patient were somehow similar to that of HO-1 deficient cases reported previously, as well as *Hmox1* knocked out mice. We speculate that the clinical manifestations of HO-1 deficient patients can be partially dependent on the type of mutation they inherit.

Hemeoxygenases (HO) are a group of enzymes involved in iron recycling(1). In humans, this family consists of two members, including HO-1 and HO-2(2). The genes encoding human HO-1 and HO-2 have been assigned to chromosome 22q12 and 16p13.3, respectively(3). The *HMOX1* gene contains ﬁve exons and encodes a 288 amino acid protein. These enzymes catalyze the oxidative conversion of heme into biliverdin, carbon monoxide, and iron(3). The constitutive expression of *HMOX2* has been demonstrated in different tissues; however,*HMOX1* has been shown to be induced in response to its substrates, including heme and heme-derived metabolites(4, 5). 

In spite of its action in heme conversion, it has also been demonstrated that HO-1 has an important antioxidant role(2, 6). In this regard, different studies have suggested that HO-1 plays a protective role in various inflammatory conditions(2, 6, 7). One of the well-known mechanisms by which HO-1 exerts antioxidant effects is the production of biliverdin. Biliverdin is then reduced and metabolized into bilirubin by another enzyme, which is a crucial endogenous antioxidant and protects tissues from damage induced by a variety of oxidative stressors(8, 9).

As the heme degradation is mainly occurring in the liver and spleen, the highest expression of *HMOX1*has also been detected in these tissues(5, 10, 11). Several studies have shown that a high level of HO-1 is produced particularly in hepatic and splenic tissue-resident macrophages in response to inflammatory mediators(5, 10-12). These macrophages have an anti-inflammatory phenotype and are the primarycells that respond to oxidative injury. Therefore, the impaired function of HO-1 or its deficiency can hamper the function of these cells, which aggravates tissue injury through an inflammatory cascade. 

Tonegawa et al. established a HO-1 deficient mice model(13). The evidence from *Hmox1*targeted mice revealed the critical importance of HO-1 in the expulsion of iron from tissue stores. Furthermore, these findings demonstrated that any defect in the iron recycling system could result in health-threatening issues(13, 14). A remarkable consistency was observed between the clinical features of HO-1 deficient patients, and those findings obtained from *Hmox1*knocked out mice. The first patient with HO-1 deficiency (OMIM#614034) has been reported by Yachie et al.(15). Serum iron deficiency and iron overload were initial findings for HO-1 deficiency. Moreover, the findings revealed that patients with HO-1 deficiency suffered severely from high fever, massive endothelial injury, abnormal coagulation system, extensive hemolysis as well as liver and kidney failure(15).

Here, we report a new case of HO-1 deﬁciency who carried a novel stop-gain mutation in the *HMOX1*coding sequence. We also performed a literature review to compare the laboratory findings, and clinical features of our patient with those patients that have been reported previously, and *Hmox1*-targeted mice.

## Case presentation

The patient was a 17-month-old girl with 9200 g weight, 71 cm height, and 46 cm head circumference who was referred to Children's Medical Center in Tehran, Iran. She was born to consanguineous Iranian parents with a birth weight of 3200 g, length of 50 cm, and head circumference of 45 cm.She was the third child of a 28-year old mother whose delivery was performed by cesarean section at week 38. The first child of this family was a girl who was found to be completely healthy, but their second child was aborted spontaneously at 20 weeks due to unknown reasons. Four admissions ensued during the eight months follow up with the chief complaints of fever, tachypnea, and signs of respiratory distress.

During the first admission, echocardiographic evaluations showed a massive pericardial effusion (PE), which was eliminated by chest tube drainage. The PE was examined for tuberculosis (TB), viral infections as well as malignancies. Based on the results, it was found to be negative in terms of TB and enterovirus (Echovirus and Coxsackievirus). Likewise, the flow cytometry results showed no phenotypic evidence of malignancies. Moreover, the results of urine culture and blood culture showed no growth. Thereafter, the patient was managed by vancomycin and cefotaxime for 10 days.Further laboratory and clinical findings showed leukocytosis, thrombocytosis, anemia, increased erythrocyte sedimentation rate (ESR) and C-reactive protein (CRP) levels, hepatomegaly, increased aspartate aminotransferase (AST), alanine aminotransferase (ALT), alkaline phosphatase (ALP), triglycerides (TG) and elevated ferritin level. Moreover, our patient was negative in terms of Epstein-Barr virus (EBV), human immunod-eficiency virus (HIV), hepatitis B virus (HBV), and hepatitis C virus (HCV) infections. These findings, along with persistent fever, have led us to consider systemic juvenile idiopathic arthritis (SJIA) as the likely diagnosis. Therefore, the treatment was continued with 30 mg/Kg/d methylprednisolone for three days. The fever was brought down, and the treatment was followed by oral prednisolone and ibuprofen tablets. Four months later, the patient was readmitted because of recurrent fever. No abnormal findings were detected on hercardiac and pulmonary auscultation. Abdominal ultrasound examination confirmed hepatomegaly, but not splenomegaly. Blood chemistry revealed leukocy-tosis, thrombocytosis, hemolytic anemia, normal ESR and CRP levels, elevated ferritin, and decreased level of fibrinogen. Then, 30 mg/Kg/d methylprednisolone was given to the patient for three days to diminish fever and resolve the symptoms. A few days later, she was discharged with the resolution of symptoms.

The patient was admitted again three months after the second admission since fever has recurred.

No histological and serological evidence was observed for arthritis. Echocardiographs and chest X-ray findings were essentially normal. Bone marrow aspiration was also performed, and no abnormality was seen. Similar to previous admission, there were increased levels of AST, 

**Fig. 1:**
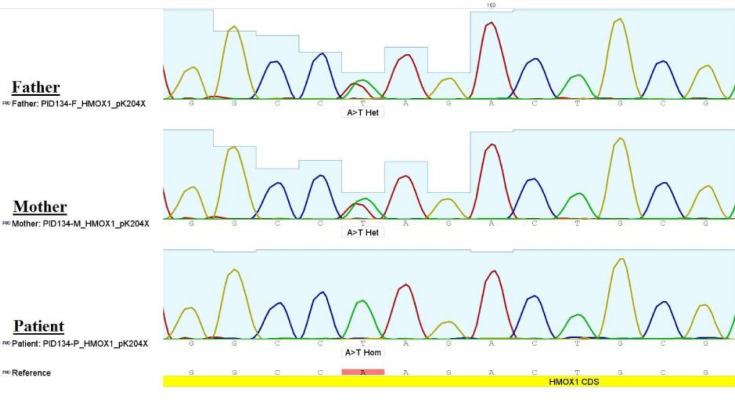
**Chromatograms of Sanger sequencing of** ***HMOX1*** **.**Whole exome sequencing (WES) identified a homozygous stop-gain mutation (exon3: c.A610T,p.K204X) on *HMOX1* coding sequence in the patient, that was confirmed by Sanger sequencing. The parents were heterozygous for the mutation

**Table 1:** Laboratory findings of the patient

**Parameter**	**Results**			
	1^st^ admission	2^nd^ admission	3^rd^ admission	4^th^ admission
**WBC (10** ^3^ **/µL)**	32.97	22.9	33.35	36
**Lymphocytes (%)**	55	59	23	34
**Neutrophils (%)**	39	35	55	65
**Hb** ** (g/dl)**	11	10.8	9.5	9.2
**Platelets (10** ^3^ **/µL)**	1115	979	1000	1000
**ESR (mm/h)**	139	25	139	25
**CRP (mg/l)**	124	10	124	32
**ALT (U/L)**	142	340	656	620
**AST (U/L)**	81	351	548	580
**ALP (U/L)**	632	1012	632	813
**LDH (U/L)**	1200	2350	15876	15350
**CPK (U/L)**	217	190	109	190
**GGT (U/L)**	77	82	90	98
**Bill T (mg/dl)**	0.4	0.4	0.7	0.8
**Bill D (mg/dl)**	0.2	0.2	0.2	0.2
**TG (mg/dl)**	482	575	723	460
**Cholesterol (mg/dl)**	195	211	213	224
**Ferritin (ng/ml)**	12870	18320	26250	27425
**Fibrinogen (mg/dl)**	142	141	125	127
**PT (s)**	14.8	14.5	15.5	17.5
**PTT (s)**	42	41	35	31
**INR**	1.2	1.1	1.4	1.6
**BUN (mg/dL)**	9	25	9	17
**Cr (mg/dL)**	0.4	0.5	0.4	0.5

WBC: white blood cell; RBC:reed blood cell; Hb: hemoglobin; ALT:alanine aminotransferase; AST:aspartate aminotransferase; ALP:alkaline phosphatase; TG:triglycerides; GGT:gamma-glutamyl transferase;HIV: human immunodeficiency virus; PPD: purified protein derivative;B/C: blood culture; U/C: urine culture; U/A: urine analysis; BUN: blood urine nitrogen; ESR: erythrocyte sedimentation rate; CRP: C-reactive protein; CPK:creatine phosphokinase;CKMB:creatine kinase-MB; PTT: partial thromboplastin time; PT: prothrombin time; INR:international normalized ratio; LDH: lactate dehydrogenase; Na: sodium; K: potassium; Bill T: total bilirubin; Bill D: direct bilirubin; Retic: reticulocyte; N: negative

ALT, ALP, TG, ferritin, ESR, CRP, and creatine phosphokinase (CPK). A liver biopsy was performed because of the persistent elevated liver function tests and hepatomegaly. The liver biopsy showed iron deposits. Similar to previous admissions, hematological analyses showed leukocytosis, thrombocytosis, and hemolytic anemia. The urine cultures were found to be positive for *Escherichia coli* (*E.coli*) sensitive to cotrimoxazole; thus a combination of prednisone and antibiotics was given to the patient. The fever was resolved within 24 h, and she was discharged.Laboratory findings in all admission are summarized in Table 1.

After five days, she was admitted once again because of a high fever. The fever and other symptoms resolved by corticosteroids and cotrimoxazole. Unfortunately, after a short remission, the clinical condition of the patient was progressively deteriorated, and she has died of recurrent fever, hemorrhage, heart failure, and ascites.

The clinical and laboratory findings (fever, hepatomegaly, iron deposits in liver cells, elevated ferritin level as well as elevated levels of liver enzymes (Table 1) have led us to suspect hemophagocyticlymphohistiocytosis (HLH) and HO-1deficiency.In this regard, to identify the genetic cause(s) responsible for this phenotype, we performed post-mortem whole exome sequencing followed by mutation confirmation by direct Sanger sequencing on the blood sample taken from the patient during the last admission when the patient was alive. Genetic testing revealed that the child carried a novel homozygous mutation (exon3: c.A610T) on the *HMOX1* gene (ENSG 00000100 292), while the parents were found to be heterozygous for that mutation (Figure 1). This is a nonsense mutation (p.K204X) which could result in HO-1 deficiency and potentially responsible for the phenotype.A search in The Human Gene Mutation Database (HGMD®) and the Online Mendelian Inheritance in Man (OMIM®) confirmed the novelty of mutation.

**Fig. 2:**
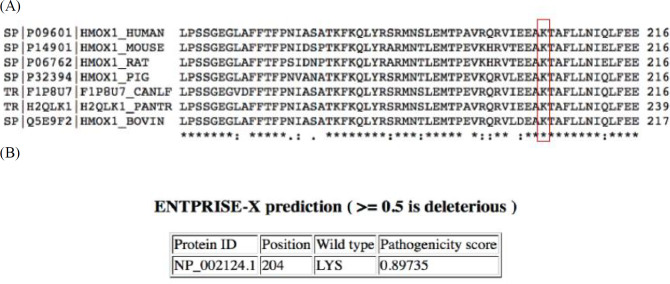
**Sequence alignment for HO-1 protein in different species.**A:HO-1 in human and corresponding region (amino acids 157-216) in six other species. The amino acid Lys204 (red) is conserved;B:ENTPRISE-X predicts that changes at codon 204 could be deleterious to the HO-1 protein

Sequence alignment (https:// www.uniprot. org/align/) showed that the K204 of HO-1 protein is conserved in several species (Figure 2A). Moreover, ENTPRISE-X (http://cssb2. biology. gatech. edu/entprise-x/) predicted that mutations at codon 204 of HO-1 are deleterious (Figure 2B). p.K204X mutation produces a truncated protein of 203 amino acids (instead of 288 amino acids) that is predicted to be highly pathologic (http:// provean. jcvi.org/ downloads. php).

## Discussion

Here, we describe a new case of HO-1 deficiency. Our patient presented with fever, leukocytosis, thrombocytosis, anemia, and increased levels of AST, ALT, ALP, TG, ferritin, ESR, CRP, and CpK (Table 1). Moreover, clinicopathological findings revealed hepatomegaly without splenomegaly and iron deposits in liver cells. Different possible underlying causes, including EBV, HIV, HBV, and HCV infections as well as other auto-inflammatory syndromes, were ruled out. Therefore, the above-mentioned features led us to consider the HO-1 deficiency as the underlying disease. Given the high fever, anemia, leukocytosis, and thrombocytosis, the patient was initiated on corticosteroids to resolve the symptoms.

According to the literature, the clinical manifestations of our patient were somehow compatible with those presentations of *Hmox1* knockout mice. Tonegawa et al. developed a mice model of HO-1 deficiency.They showed that the lack of a functional HO-1 could result in pathologic iron deposition in liver cells, and increased sensitivity to oxidative stress(13). The mice was also characterized by hepatomegaly, asplenia, anemia, leukocytosis, thrombocytosis, increased ferritin level, and growth retardation.

To the best of our knowledge, there are only two previous human case reports on the HO-1 deficiency. The first human case of HO-1) deficiency was reported by Yachie et al. (15). Their patient was a six-year old boy. Similar to our patient and mice lacking HO-1, they observed hepatomegaly with iron deposits in the liver and renal tissues of their patient. Recurrent fever, persistent hemolytic anemia, leukocytosis, abnormal coagulation system as well as elevated lactate dehydrogenase (LDH), AST, and ferritin were the other characteristics that were common between our patient and their patient. Hyperlip-idemia was the other same finding between these two cases. Yachie et al. have found that complete loss of exon 2 in the maternal allele and a two-nucleotide deletion within exon 3 in the paternal allele were responsible for the phenotype (15).

Kawashima et al.performed post-mortem autopsy on the patient described by Yachie et al.(16). Their findings showed asplenia, deposition of iron pigments in liver macrophages, and amyloid deposits in the adrenal and hepatic tissues(16). 

Although asplenia was seen inthe HO-1 deficient patient reported by Yachie et al., our patient exhibited normal spleen size. We assume that different mutations in *HMOX1* may have no effect on spleen mass or can result in asplenia or splenomegaly. In this regard, Greil et al. reported a patient carrying G139V mutation in the *HMOX1* who had splenomegaly(17, 18). Moreover, previous studies have assumed that asplenia may contribute to intravascular survival of damaged cells, thus further increase anemia and oxidative stress(15, 19). Since our patient had normal spleen mass, the mentioned speculation may not be accurate. Instead, we assume that HO-1 deficiency may hamper the function of splenocytes, thus resulting in augmented oxidative stress. 

Indeed, HO-1 is induced under inflammatory conditions and plays an essential role in the resolution of oxidative stress as an endogenous antioxidant(2). Thus, the lack of HO-1 or its impaired function may result in disseminated intravascular hemorrhage, which subsequently leads to patient’s death.

The second patient with HO-1 deficiency was described recently by Radhakrishnan et al.(20). Their patient was a fifteen-year old female whose chief complaint was high fever. The disease cause was found to be a homozygous missense mutation in exon 2 (R44X) of the *HMOX1* coding sequence. Similar to the case reported by Yachie et al., she was also presented with leukocytosis, thrombo-cytosis, elevated AST, ALT, LDH, ferritin, and asplenia. Consistent with our patient, the patient described by Radhakrishnan et al. was refractory to corticosteroids. 

We noticed that our patient and the case that was reported by Yachie et al. have manifested the disease in their infantile period and died in early years of their lives; however, Radhakrishnan’s patient was diagnosed and died 15 years of her life. It seems that other compensatory mechanisms, likely HO-2, might exist behind this inconsistency, or the proper function of HO-1 is more important at the older ages. However, further investigation should be done on appropriate animal models to shed light on this complex issue. 

In conclusion, we report a new case of HO-1 deficiency presenting with HLH like presentation. The phenotype of our patient was compatible with those two patients reported previously. In contrast to previous HO-1 deficient cases who exhibited asplenia, our patient has normal spleen mass. This case shows that there may be a partial difference between the pathophysiological findings of the cases with the same disease, which might result from the type of mutation they inherit.

## Consent

Written informed consent was obtained from the parents of the patient for the publication of this case report.

## Conflict of interest

The authors declare that they have no Compe-ting interests.
